# Determinants of cigarette smoking and smoking frequency among women of reproductive age in Nigeria: evidence from a nationwide cross-sectional survey

**DOI:** 10.1186/s13011-023-00530-5

**Published:** 2023-04-05

**Authors:** Daniel Chukwuemeka Ogbuabor, Alphonsus Ogbonna Ogbuabor, Matthew Chibunna Igwe

**Affiliations:** 1 Department of Health Administration and Management, University of Nigeria, Enugu Campus, Enugu, Enugu State Nigeria; 2 Department of Health Systems and Policy, Sustainable Impact Resource Agency, Enugu, Nigeria; 3 Department of Medical Laboratory Sciences, College of Medicine, Enugu State University of Science and Technology, Enugu, Nigeria

**Keywords:** Cigarette smoking, Smoking behaviour, Women, Risk factors, Demographic and health survey, Nigeria

## Abstract

**Background:**

Smoking is a leading cause of avoidable deaths and attributable disability-adjusted life years globally. Yet, the determinants of smoking practices among women are understudied. This study assessed the determinants of smoking and smoking frequency among women of reproductive age in Nigeria.

**Methods and materials:**

Data from the 2018 Nigeria Demographic and Health Survey (NDHS) were used in this study (*n* = 41,821). The data were adjusted for sampling weight, stratification, and cluster sampling design. The outcome variables were smoking status and smoking frequency (daily smoking and occasional smoking). The predictor variables included women’s socio-demographic and household characteristics. Pearson’s chi-squared test was used to evaluate the association between outcome and predictor variables. All variables significant in bivariate analyses were further analysed using complex sample logistics regression. Statistical significance was set at a *p*-value < 0.05.

**Results:**

The prevalence of smoking among women of reproductive age is 0.3%. The prevalence of smoking frequency is 0.1% (daily) and 0.2% (occasionally). Overall, women aged 25-34 (AOR = 2.13, 95%CI: 1.06-4.29, *ρ* = 0.034), residing in the South-south region (AOR = 9.45, 95%CI: 2.04-43.72, *ρ* <0.001), being formerly married (AOR = 3.75, 95%CI: 1.52-9.21, *ρ* = 0.004), in female-headed households (AOR = 2.56, 95%CI: 1.29-5.08, *ρ* = 0.007) and owning mobile phones (AOR = 2.10, 95%CI: 1.13-3.90, *ρ* = 0.020) were more likely to smoke. Whereas female-headed households (AOR = 4.34, 95%CI: 1.37-13.77, *ρ* = 0.013) and being formerly married (AOR = 6.37, 95%CI: 1.67-24.24, *ρ* = 0.007) predisposed to daily smoking, age 15-24 (AOR = 0.11, 95%CI: 0.02-0.64, *ρ* = 0.014) was protective of daily smoking among women. Owning mobile phones (AOR = 2.43, 95%CI: 1.17-5.06, *ρ* = 0.018) increased the odds of occasional smoking among women.

**Conclusions:**

The prevalence rates of smoking and smoking frequency are low among women of reproductive age in Nigeria. Women-centred approaches to tobacco prevention and cessation must become evidence-informed by incorporating these determinants into interventions targeting women of reproductive age in Nigeria.

## Background

Cigarette smoking is a persisting global health concern and remains a leading risk factor for attributable disability-adjusted life-years (DALYs) and avoidable deaths [1]. Cigarette smoking accounted for 7.69 million deaths and 200 million DALYs, constituting 13.6% of all human deaths and 7.89% of all DALYs in 2019 [2]. Smoking increases the incidence of infections and aggravates the progress and prognosis of infectious diseases in a dose-dependent manner [3]. Cigarette smoking among women predisposes them to non-communicable diseases, including cancer, heart disease, stroke, chronic respiratory diseases, and diabetes [4–7]. The risk of smoking-related lung cancer in women may eventually exceed those of men, once cumulative exposure to smoking in women is comparable to that in men [8]. Cigarette smoking also negatively impacts pregnancy and the health of unborn children of women of reproductive age [4, 6, 7, 9, 10]. Further, the cost of cigarette consumption can also contribute to household poverty [11, 12]. Yet, research on women-centred approaches to the control of smoking other than pregnancy and smoking is scarce [13].

Despite decreasing global trends, cigarette smoking is increasing among women of reproductive age (WRA) in high-income countries (HICs) [14]. Cigarette smoking among women in low- and middle-income countries (LMICs) is lower than HICs because LMICs are earlier in the epidemiological transition of tobacco use [9]. Yet, tobacco industry products and marketing increasingly target women in LMICs resulting in not only a disproportionately slower decrease in tobacco smoking in women than men but also a gradually increasing trend that might significantly shift the global lower prevalence of smoking among women in LMICs [4, 15]. The 32% pooled prevalence of ever cigarette smoking in women in Africa is higher than the global prevalence of 28% [16]. In Nigeria, the pooled prevalence of ever smoking in women is 6.3% is concerning since smoking prevalence is rising at about 4% per annum [17].

Evidence from published studies on the prevalence of cigarette smoking in women is mixed. In LMICs, the prevalence of smoking was 0.69% among pregnant women and 1.09% among nonpregnant women [9]. The prevalence rate was 0.18% in Kenya [18]. Smoking prevalence rates among women were low in Sub-Saharan Africa, Eastern Mediterranean and East Asia regions (<10%), and relatively high in Eastern Europe, Latin America, and Southeast Asia (15%-21%) [19]. The prevalence rates ranged from 2 to 3 per cent in Senegal, Congo, Thailand, and China [20, 21]; 5 to 7 per cent in Iran, Kazakhstan, and Pakistan [5, 19]; and 12 to19 per cent in Iran, Mexico, South Africa, India, Brazil, United States of America, and Viet Nam [5, 6, 14, 19, 22–25].

On the determinants of cigarette smoking among women, evidence indicates that familial and partner influences [20], low income [4, 5, 7, 9, 21, 22, 26, 27], low education [4, 7, 9, 18, 21, 22, 28, 29], greater education [30, 31], urban areas [5, 7, 21], rural areas [10], older age [5, 18, 19, 27], younger age [26], being married [5], being formerly married (divorced/separated/widowed) [18, 27], female-headed household [29], being employed [7], unemployment [4], perceiving distance as a problem in seeking healthcare [7], religion [27], severely food insecure women [32], depression and stress [26], narcissistic and impulsive personality traits [28], region heterogeneity [7, 21], multiparity [4], television viewing [23], and intimate partner sexual violence [33] increased the likelihood of cigarette smoking among women of reproductive age.

There is a knowledge gap on the prevalence and determinants of cigarette smoking among women in Sub-Saharan Africa, with most research in this area conducted in high-income and other LMICs. Most studies in Nigeria did not use nationally representative samples and the samples also included men [17]. This study, therefore, fills an important gap by providing evidence of the prevalence and determinants of cigarette smoking and smoking frequency among women of reproductive age using a nationally representative sample. Such evidence would be useful to health decision-makers and practitioners in developing more inclusive and integrated women-centred strategies for the control of cigarette smoking and reducing the associated consequences for women of reproductive age in Nigeria.

## Methods

### Study setting

The study was conducted in Nigeria in 2018. Women of reproductive age constituted about 46% of the estimated Nigerian population of 195,874,683 people in 2018 [34]. The population is growing at about 3% [34]. Nigeria consists of 36 states and a Federal Capital Territory, which are delineated into six geopolitical regions. Each state consists of local government areas (LGAs). Each LGA is composed of wards. The wards are further delineated into enumeration areas (EAs), described as clearly defined geographic areas that group several households together for population and housing census [35].

### Study design

We conducted a secondary analysis of data from the Nigeria Demographic and Health Survey (NDHS) 2018 based on a cross-sectional, household survey design.

### Sampling strategy

Using an estimated proportion of WRA that are anaemic (*P* = 0.578), design effect (Deft = 1.434), relative standard error (*α*= 0.01), individual response rate (*R*_*i*_ = 97%), household gross response rate (*R*_*h*_ = 95%), and the number of eligible individuals per household (d = 1.032) [35], the sample size in terms of the number of households (n) was calculated using the formula [36]:

$$n={\mathrm{Deft}}^{2}\times \frac{\left(1/P-1\right)}{{\alpha }^{2}}/\left({R}_{i}\times {R}_{h}\times d\right)$$

The sample size was 42,000 households. A two-stage stratified sampling technique was used to select the households. The sampling frame consisted of households listed in Nigeria’s 2006 Population and Housing Census (NPHC). The primary sampling unit (PSU) is a cluster of enumeration areas (EAs). Each of the thirty-six states and the Federal Capital Territory was stratified into urban and rural areas, creating 74 sampling strata. In the first stage, 1,400 (580 urban and 820 rural) EAs were selected from the sampling strata with probability proportional to EA size. In the second stage’s selection, 30 households were selected from every cluster through equal probability systematic sampling, resulting in a total sample size of about 42,000 households.

### Data collection

**Fig. 1:**
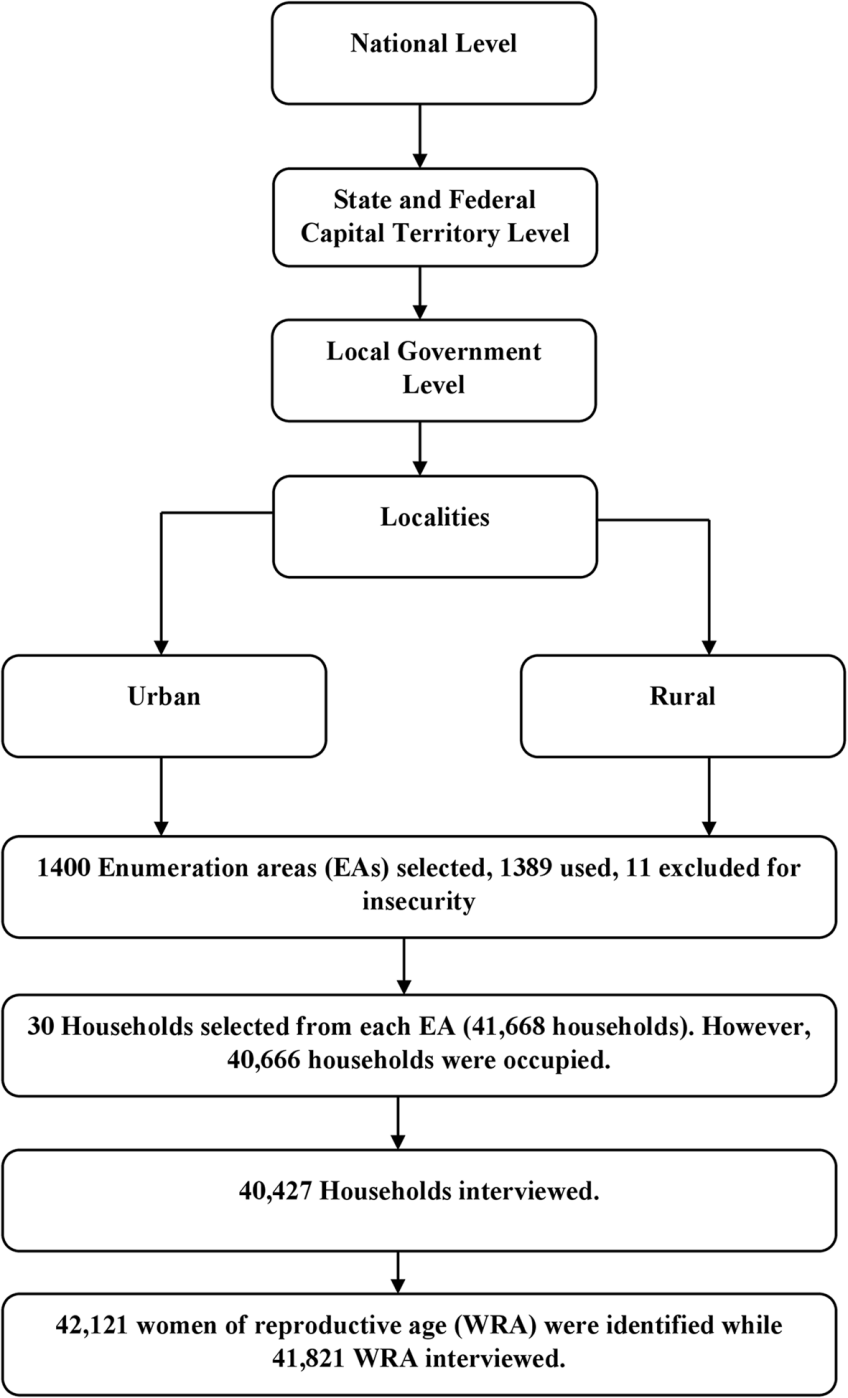
Flow diagram for the study sample

The survey was successfully carried out in 1,389 clusters in 36 states and Federal Capital Territory comprising 747 LGAs from August to December 2018 through one-on-one interviews. A total of 41,668 households were selected for the sample, of which 40,666 were occupied (Fig. 1). Of the occupied households, 40,427 were successfully interviewed, yielding a response rate of 99%. In the households interviewed, 42,121 women aged 15-49 were identified for individual interviews; interviews were completed with 41,821 women, yielding a response rate of 99%. No incentives were offered to women for participating in the study. Eleven clusters, with deteriorating law-and-order situations, were dropped during the fieldwork. To prevent bias, no replacements and no changes to the pre-selected households were allowed in the implementing stages. The inclusion criteria were all WRA, either permanent residents or visitors who stayed in the sampled household the night before the survey. Women who did not agree to provide consent and women outside the age of 15-49 years were excluded. The study was also limited to combustible cigarettes.

### Variables

#### Outcome variable

The outcome variables were smoking status and smoking frequency at the time of the survey. In this study, smoking cigarettes were limited to smoking combustible cigarettes including manufactured cigarettes and hand-rolled cigarettes [35]. Cigars, cheroots, cigarillos, hookah, kreteks, and e-cigarettes were excluded. The specific question that we used to assess current cigarette use was "Do you currently smoke cigarettes?" Those who responded ‘yes’ to this question were defined as women who currently smoke cigarette, whereas those who responded ‘no’ were defined as women who do not currently smoke. Missing data on whether smoked cigarettes were assumed to be non-use of cigarettes and were excluded from the numerator but included in the denominator. Regarding smoking frequency, women were further asked whether they smoked daily or some days. The smoking frequency was recoded into a binary variable as ‘every day’ (daily) and ‘some days’ (occasional) implying smoking sometimes, but less than daily [35].

#### Predictor variables

The predictor variables were selected based on background knowledge, existing literature, and availability of data in the DHS database. The variables were grouped into individual characteristics and socio-economic and household characteristics. The individual characteristics included age, marital status (Never in a union, married/living with a partner, and divorced/separated/widowed), sex of household head (female and male), religion (Catholic, Other Christians, Islam, and Others), pregnancy status (no/unsure, yes), and gender attitude (good, bad). Gender attitudes were measured using five variables describing women’s attitudes towards domestic violence including whether beating was justified if the wife goes out without telling her husband; neglects the children; argues with her husband; refuses sex with her husband; and burns food [37]. Women who answered ‘Yes’ and ‘Don’t know’ were coded as 1, while women who responded ‘No’ were coded as 0. Women were categorized into good gender attitude if they answered ‘No’ to all five variables, and poor gender attitude if they answered ‘Yes’ or ‘Don’t know’ to any of the five questions. The socioeconomic and household characteristics included region (North-Central, North-East, North-West, South-East, South-South, and South-West), type of residence (urban and rural), highest education (no education, primary, secondary, and higher), employment (unemployed and employed), wealth index (poorest, poor, moderate, rich, richest), radio exposure (not at all, yes), television exposure (not at all, yes), ownership of mobile phone (yes, no), internet use (yes, no), and literacy (illiterate and literate). Regarding the wealth index, households were given scores, derived using principal component analysis, based on the number and kinds of consumer goods they own, ranging from a television to a bicycle or car, and housing characteristics such as the source of drinking water, toilet facilities, and flooring materials [35]. The wealth quintiles were compiled by assigning the household score to each usual household member, ranking each person in the household population by her score, and then dividing the distribution into five equal categories, each comprising 20% of the population [35]. We, however, regrouped the wealth index into three categories poor (poorest and poor), moderate, and rich (rich and richest). Mental health was not included as a predictor variable since the DHS did not collect data on women’s mental health.

### Statistical analysis

Statistical analyses were carried out on SPSS 20 (IBM Corp., Armonk, NY). To account for the non-proportional allocation of the sample to the different states and provide representative population estimates, the data were adjusted for sampling weights, stratification, and multistage sampling before analysis. The independent variables did not show any multicollinearity given that the variance inflation factors (VIF) ranged from 1.04 - 3.79. We summarized women’s basic characteristics using frequencies, population estimates and percentages (weighted). The sampling weights were calculated based on sampling probabilities separately for each sampling stage and each cluster. The individual women’s sampling weight was derived from household sampling weight multiplied by the inverse of women’s individual response rate by stratum. Individual sample weights were generated by dividing (v005) by 1,000,000 before analysis to approximate the number of cases. The Chi-square test was used to determine the association between smoking and smoking frequency and independent variables. All significant predictor variables in the Chi-square analyses were included as covariates in the multivariable complex sample logistic regression model to determine the adjusted effect of each predictor variable on the outcome variables. The results of regression analysis were presented by crude/unadjusted odds ratio (COR) and adjusted odds ratio (AOR) with 95% confidence intervals (CIs) and *p*-values. Statistical significance for all analyses was set at *p* <0.05. The model fit was tested using McFadden’s r-squared because it compares the likelihood-ratio of the current model to a model without any covariates and represents the amount of variation explained by the current model [38]. The McFadden test statistic for smoking status and smoking frequency was 0.1 and 0.2 respectively. Since values ranging from 0.2 to 0.4 indicate a good model fit, the model for smoking frequency represents a better fit relative to the model for smoking status [38].

### Ethical consideration

We did not obtain further ethical approval as this was a secondary data analysis. In the primary study, ethical approval was obtained from the National Health Research Ethics Committee of Nigeria (NHREC) and the ICF Institutional Review Board. Additionally, informed consent was obtained from participants before interviews were conducted.

## Results

### Characteristics of respondents

The basic characteristics of the respondents are shown in Table 1. Most women were married/living with a partner and lived in a male-headed household. About 54% of WRA reside in urban areas. Almost 35% of WRA had no education. The proportion of women of reproductive age (WRA) from the regions ranged from 11.6% (South-East) to 29.2% (North-West). Over 50% of women were literate, had media exposure or owned a mobile phone.

**Table 1:** Basic characteristics of women of reproductive age in the study sample (*n* = 41821) in Nigeria, 2018

Characteristics		n	%
Age group	15-24	15284	36.5
	25-34	13433	32.1
	35-49	13105	31.3
Region	North-Central	5891	14.1
	North-East	6636	15.9
	North-West	12225	29.2
	South-East	4963	11.9
	South-South	4840	11.6
	South-West	7266	17.4
Type of place of residence	Urban	19163	45.8
	Rural	22658	54.2
Highest educational level	No education	14603	34.9
	Primary	6039	14.4
	Secondary	16583	39.7
	Higher	4596	11.0
Current marital status	Never in union	10550	25.2
	Married/LP	29090	69.6
	Divorced/Separated/Widowed	2181	5.2
Religion	Catholic	4345	10.4
	Other Christian	14872	35.6
	Islam	22372	53.5
	Other	232	.6
Sex of household head	Male	34891	83.4
	Female	6930	16.6
Literacy	Illiterate	19630	47.0
	Literate	22191	53.0
Radio exposure	Not at all	18478	44.2
	Yes	23343	55.8
TV exposure	Not at all	19992	47.8
	Yes	21829	52.2
Owns a mobile telephone	No	18688	44.7
	Yes	23133	55.3
Internet use	Not at all	35503	84.9
	Yes	6318	15.1
Wealth index	Poor	15400	36.8
	Middle	8572	20.5
	Rich	17849	42.7
Employment status	No	14645	35.0
	Yes	27176	65.0
Current pregnant status	No or unsure	37585	89.9
	Yes	4236	10.1
Gender attitude	Good	29728	71.1
	Poor	12093	28.9

### Prevalence of smoking

Overall, about 0.3% (95% CI: 0.2–0.4) of WRA smoke. Smoking prevalence among women significantly differed with age, region, marital status, sex of household head, ownership of a mobile phone, and employment status (Table 2).

**Table 2:** Prevalence of smoking among women of reproductive age (*N* = 41821), Nigeria, 2018

		Smokes cigarettes					
		No		Yes			
		n	Per cent (%)	n	Per cent (%)	X^2^	*p*-value
Age group	15-24	15257	99.8	27	0.2		
	25-34	13375	99.6	57	0.4	19.7	0.009^*^
	35-49	13078	99.8	27	0.2		
Region	North-Central	5872	99.7	18	0.3		
	North-East	6619	99.7	17	0.3	50.8	<0.001^*^
	North-West	12209	99.9	16	0.1		
	South-East	4961	100.0	1	0.0		
	South-South	4826	99.7	14	0.3		
	South-West	7222	99.4	44	0.6		
Type of place of residence	Urban	19098	99.7	64	0.3	6.4	0.131
	Rural	22612	99.8	47	0.2		
Highest educational level	No education	14582	99.9	21	0.1		
	Primary	6022	99.7	16	0.3		
	Secondary	16525	99.6	58	0.4	14.2	0.060
	Higher	4581	99.7	16	0.3		
Religion	Catholic	4335	99.8	10	0.2		
	Other Christian	14815	99.6	57	0.4	15.1	0.066
	Islam	22330	99.8	42	0.2		
	Other	230	99.3	2	0.7		
Current marital status	Never in union	10504	99.6	46	0.4		
	Married/LP	29046	99.8	44	0.2	65.3	<0.001^*^
	Formerly	2160	99.1	21	0.9		
Sex of household head	Male	34834	99.8	57	0.2	80.3	<0.001^*^
	Female	6876	99.2	53	0.8		
Literacy	Illiterate	19578	99.7	52	0.3	0.001	0.989
	Literate	22132	99.7	59	0.3		
Radio	Not at all	18422	99.7	57	0.3	2.2	0.407
	Yes	23289	99.8	54	0.2		
TV	Not at all	19955	99.8	37	0.2	9.6	0.052
	Yes	21755	99.7	74	0.3		
Own a mobile phone	No	18666	99.9	21	0.1	28.9	<0.001^*^
	Yes	23044	99.6	89	0.4		
Internet	Not at all	35416	99.8	87	0.2	3.8	0.204
	Yes	6294	99.6	24	0.4		
Wealth index	Poor	15364	99.8	36	0.2		
	Middle	8543	99.7	29	0.3	2.2	0.689
	Rich	17803	99.7	46	0.3		
Employment	No	14623	99.9	22	0.1	11.7	0.011^*^
	Yes	27087	99.7	89	0.3		
Currently pregnant	No or unsure	37486	99.7	99	0.3	0.1	0.795
	Yes	4224	99.7	12	0.3		
Gender attitude	Good	29640	99.7	88	0.3	3.9	0.108
	Poor	12070	99.8	23	0.2		
Total	Total	41710	99.7	111	0.3		

^*^Significant at *p* < 0.05, Chi-square test

### Prevalence of frequency of smoking

The prevalence of daily smoking was 0.1% (95% CI: 0.1–0.2) while the prevalence of occasional smoking was 0.2% (95% CI: 0.1–0.2). The prevalence of smoking frequency significantly varied with age, education, marital status, sex of household head, ownership of a mobile phone, internet use, and employment status (Table 3)

**Table 3:** Prevalence of smoking frequency among women of reproductive age, Nigeria, 2018

Characteristics		Frequency smokes cigarettes							
		Does not smoke		Every day		Some days			
		n	Per cent (%)	n	Per cent (%)	n	Per cent (%)	X^2^	*P*-value
Age groups	15-24	15257	99.8	2	0.0	24	0.2	32.3	0.002*
	25-34	13375	99.6	30	0.2	28	0.2		
	35-49	13078	99.8	11	0.1	16	0.1		
Region	North-Central	5872	99.7	5	0.1	13	0.2		
	North-East	6619	99.7	6	0.1	11	0.2	52.8	0.060
	North-West	12209	99.9	8	0.1	8	0.1		
	South-South	4961	100.0			1	0.0		
	South-West	4826	99.7	7	0.1	7	0.2		
	South-East	7222	99.4	18	0.2	26	0.4		
Type of place of residence	Urban	19098	99.7	23	0.1	41	0.2	7.1	0.340
	Rural	22612	99.8	20	0.1	26	0.1		
Highest educational level	No education	14582	99.9	4	0.0	17	0.1		
	Primary	6022	99.7	6	0.1	10	0.2	22.3	0.046*
	Secondary	16525	99.6	30	0.2	29	0.2		
	Higher	4581	99.7	4	0.1	12	0.3		
Religion	Catholic	4335	99.8	5	0.1	6	0.1		
	Other Christian	14815	99.6	23	0.2	34	0.2	18.6	0.338
	Islam	22330	99.8	16	0.1	26	0.1		
	Other	230	99.3			2	0.7		
Current marital status	Never in union	10504	99.6	19	0.2	27	0.3		
	Married/LP	29046	99.8	11	0.0	33	0.1	84.4	<0.001*
	Formerly	2160	99.1	13	0.6	8	0.4		
Sex of household head	Male	34834	99.8	13	0.0	44	0.1	103.7	<0.001*
	Female	6876	99.2	30	0.4	23	0.3		
Literacy	Illiterate	19543	99.7	27	0.1	24	0.1		
	Literate	22132	99.7	16	0.1	43	0.2	19.1	0.106
	Other	35	97.6			1	2.4		
Radio	Not at all	18422	99.7	28	0.1	29	0.2	6.6	0.292
	Yes	23289	99.8	16	0.1	38	0.2		
TV	Not at all	19955	99.8	15	0.1	21	0.1	9.7	0.190
	Yes	21755	99.7	28	0.1	46	0.2		
Owns a mobile telephone	No	18666	99.9	8	0.0	14	0.1	29.0	<0.001*
	Yes	23044	99.6	36	0.2	53	0.2		
Internet use	Not at all	35416	99.8	40	0.1	47	0.1	14.0	0.020*
	Yes	6294	99.6	4	0.1	20	0.3		
Wealth index	Poor	15364	99.8	10	0.1	26	0.2		
	Middle	8543	99.7	20	0.2	8	0.1	21.5	0.093
	Rich	17803	99.7	13	0.1	33	0.2		
Employment	No	14623	99.9	6	0.0	16	0.1	12.7	0.021*
	Yes	27087	99.7	38	0.1	52	0.2		
Currently pregnant	No or unsure	37486	99.7	40	0.1	58	0.2	1.2	0.660
	Yes	4224	99.7	3	0.1	9	0.2		
Gender attitude	Good	29640	99.7	34	0.1	54	0.2	4.0	0.307
	Poor	12070	99.8	10	0.1	13	0.1		
Total	Total	41710	99.7	44	0.1	67	0.2		

^*^Significant at *p* < 0.05

### Determinants of smoking among women of reproductive age

Age 25-35 years (AOR = 2.13, 95%CI: 1.06-4.29, *ρ* = 0.034), residing in the South-South region (AOR = 9.45, 95%CI: 2.04-43.72, *ρ* <0.001), being divorced/separated/widowed (AOR = 3.75, 95%CI: 1.52-9.21, *ρ* = 0.004), female sex of household head (AOR = 2.56, 95%CI: 1.29-5.08, *ρ* = 0.007), and ownership of mobile phone (AOR = 2.10, 95%CI: 1.13-3.90, *ρ* = 0.020) significantly increased the odds of smoking among WRA (Table 4).

**Table 4:** Factors associated with the odds of smoking among women of reproductive age, Nigeria, 2018

Characteristics			95% Confidence Interval				95% Confidence Interval		
		Odds Ratio	Lower	Upper	*P*-value	Adjusted Odds Ratio	Lower	Upper	*P*-value
Age groups	15-24	0.65	0.22	1.90	0.429	0.56	0.19	1.64	0.288
	25-34	2.21	1.10	4.48	0.027*	2.13	1.06	4.29	0.034*
	35-49	1.00				1.00			
Region	North-Central	16.31	3.72	71.45	0.413	16.41	3.75	71.85	0.395
	North-East	18.09	3.87	84.53	0.613	17.64	3.77	82.41	0.547
	North-West	12.30	2.74	67.40	0.252	11.40	2.09	62.12	0.188
	South-South	9.40	2.03	43.46	<0.001*	9.45	2.04	43.72	<0.001*
	South-West	22.48	4.95	102.16	0.079	22.86	5.03	103.85	0.076
	South-East	1.00				1.00			
Current marital status	Never in union	3.71	1.43	9.60	0.973	3.53	1.38	9.03	0.928
	Divorced/Separated/Widowed	3.79	1.54	9.33	0.004*	3.75	1.52	9.21	0.004*
	Married/Living in a union	1.00				1.00			
Sex of household head	Female	2.49	1.26	4.90	0.008*	2.56	1.29	5.08	0.007*
	Male	1.00				1.00			
Owns a mobile telephone	Yes	2.00	1.08	3.70	0.028*	2.10	1.13	3.90	0.020*
	No	1.00				1.00			
Employment	Yes	1.71	0.87	3.38	0.121				
	No	1.00							

*Significant at *p* value < 0.05, *OR* Odd ratio, *AOR* Adjusted odd ratio

### Determinants of daily smoking among women of reproductive age

Being divorced/separated/widowed (AOR = 6.37, 95%CI: 1.67-24.24, *ρ* = 0.007), and female head of household (AOR = 4.34, 95%CI: 1.37-13.77, *ρ* = 0.013) significantly increased the odds of smoking among WRA (Table 5). In contrast, ages 15-24 years (AOR = 0.11, 95%CI: 0.02-0.64, *ρ* = 0.014) significantly reduced the likelihood of daily smoking among WRA (Table 5).

**Table 5:** Factors associated with the odds of daily smoking among women of reproductive age, Nigeria, 2018

Characteristics			95% Confidence Interval				95% Confidence Interval		
		OR	Lower	Upper	*P*-value	AOR	Lower	Upper	*P*-value
Age groups	15-24	0.09	0.02	0.57	0.010*	0.11	0.02	0.64	0.014*
	25-34	2.99	0.87	10.25	0.082	3.21	0.96	10.78	0.059
	35-49	1.00				1.00			
Highest educational level	No education	0.62	0.13	2.95	0.547				
	Primary	1.14	0.29	4.48	0.852				
	Secondary	2.03	0.61	6.75	0.250				
	Higher	1.00							
Current marital status	Never in union	8.51	2.08	34.84	0.773	9.52	2.33	38.84	0.693
	Formerly	6.37	1.69	24.01	0.006*	6.37	1.67	24.24	0.007*
	Married/living in a union	1.00				1.00			
Sex of household head	Female	3.84	1.26	11.70	0.018*	4.34	1.37	13.77	0.013*
	Male	1.00				1.00			
Owns a mobile telephone	Yes	1.40	0.55	3.53					
	No	1.00							
Internet use	Yes	0.18	0.04	0.88	0.034*	0.15	0.02	1.10	0.062
	Not at all	1.00							
Employment	Yes	1.75	0.55	5.51	0.340				
	No	1.00							

^*^Significant at *p* value < 0.05, *OR* Odd ratio, *AOR* Adjusted odd ratio

### Determinants of occasional smoking among women of reproductive age

Ownership of mobile phones (AOR = 2.43, 95%CI: 1.17-5.06, *ρ* = 0.018) significantly increased the odds of occasional smoking among WRA (Table 6).

**Table 6:** Factors associated with the odds of occasional smoking among women of reproductive age, Nigeria, 2018

Characteristics			95% Confidence Interval				95% Confidence Interval		
		OR	Lower	Upper	*P*-value	AOR	Lower	Upper	*P*-value
Age in 5-year groups	15-24	1.35	0.44	4.16	0.603				
	25-34	1.89	0.88	4.03	0.100				
	35-49	1.00							
Highest educational level	No education	1.83	0.56	5.98	0.316				
	Primary	1.55	0.36	6.70	0.559				
	Secondary	1.04	0.37	2.95	0.937				
	Higher	1.00							
Current marital status	Never in union	2.32	0.79	6.80	0.987				
	Formerly	2.29	0.77	6.81	0.137				
	Married/living in a union	1.00							
Sex of household head	Female	1.63	0.73	3.63	0.231				
	Male	1.00							
Owns a mobile telephone	Yes	2.70	1.42	5.14	0.002*	2.43	1.17	5.06	0.018*
	No	1.00				1.00			
Internet use	Yes	1.54	0.57	4.18	0.394				
	No	1.00							
Employment	Yes	1.74	0.74	4.08	0.201				
	No	1.00							

^*^Significant at *p* value < 0.05, *OR* Odd ratio, *AOR* Adjusted odd ratio

## Discussion

The purpose of this study was to assess the prevalence and determinants of cigarette smoking and smoking frequency among women of reproductive age in Nigeria. We found a low prevalence of cigarette smoking and smoking frequency. Older age, residing in the South-south region, being formerly married (divorced/separated/widowed), female-headed household, and mobile phone ownership influenced women’s smoking status.

Our finding of a low prevalence rate of cigarette smoking among women of reproductive age is consistent with evidence from Kenya, and a study of 42 low-and middle-income countries (LMICs) [9, 18]. In contrast, relatively higher prevalence rates were found in other African countries, Eastern Europe, Latin America, and Southeast Asia [5, 19–24]. The differing prevalence is related to the stage of the epidemic in each country [9]. Globally, female prevalence data suggest that 40% of countries, half of them in sub-Saharan Africa, are still in the incipient stage of the epidemic, with very low prevalence including Nigeria [39]. Given that the tobacco industry products and marketing increasingly target women in LMICs [4, 15], interventions to prevent and reduce smoking are needed to avert a significant increase in the prevalence of smoking among women in Nigeria. In Nigeria, a tobacco control legislation exists, which established a tobacco control unit at the Federal Ministry of Health (FMOH) and prescribed some public health measures to control tobacco use [40, 41]. However, public awareness of this regulatory framework and public health measures is low [41]. Nigeria currently has a text-only health warning requirement to cover 50% of the front and back surfaces of cigarette packages, while a pictorial warning will be included beginning in June 2024 [40–42]. Even then, the textural health warnings are provided only in the English language and should be extended to widely spoken indigenous languages as has been argued in prior studies [41, 42]. The textural warnings have also not been regularly revised and fall short of highlighting specific health effects of smoking [40]. Moreover, there is a need for immediate implementation of pictorial health warnings on cigarette packages [40]. As allowing communication with consenting persons aged 18 and above enhances the targeting of young women by the tobacco industry, Nigeria might also consider a ban on one-on-one communication with consenting adults as is the case in South Africa [41].

Additionally, to keep the current low prevalence of smoking among WRA in check, three regulatory reforms are warranted in Nigeria. First, subjecting FMOH’s tobacco regulations to approval by Nigeria’s parliament creates opportunities for tobacco lobbyists to weaken the effectiveness of tobacco control legislation [40, 41]. Secondly, the Manufacturers’ Association of Nigeria (MAN) should be excluded from membership of the National Tobacco Control Committee (NATOCC). The inclusion of MAN in NATOCC’s membership limits the effectiveness of the committee to advise the FMOH on the development and implementation of tobacco control policies since the tobacco industry is a member of MAN [40, 41]. Thirdly, the tobacco industry should not be allowed a formal role in defining its regulation through the Standard Organisation of Nigeria (SON) as this undermines the capacity of SON to regulate the content and emissions of cigarettes, especially with the exclusion of the FMOH on SON’s governing body [41, 42].

We found that while age 25-34 increased the odds of female cigarette smoking, age 15-24 reduced their likelihood of smoking daily. The findings of the current study are consistent with the evidence from previous studies [5, 18, 19, 27], but differ from one study in which younger ages were predisposed to smoking [26]. As explained in a prior study, low prevalence among young women might be related to the effect of the ban on advertising and the increase in tobacco taxes [18]. Older women might have more spending power and a higher prevalence rate because, during their youth, advertising and branding were allowed making initiation easier [18]. Our demographic findings, therefore, provide important lessons for Nigeria’s tobacco control policies. First, the principle of prevention would be to target women before the age of 25 years. Secondly, smoking cessation interventions should target women in the 25-34 age group. Arguably, the Nigerian tax rate of 28% is much lower than the WHO recommendation of 70% for tobacco taxation [40, 42]. Given that 40% of Nigerians are poor [43], increasing tobacco taxation might be a potent measure to further limit its use among women. Such a combination of strategies would help keep the prevalence of female smoking low.

Consistent with region heterogeneity found in prior studies in Nepal and China [7, 21], the current study revealed significant regional variation in smoking prevalence among women in South-south Nigeria. Residing in the South-south region increased the chances of smoking among WRA possibly due to environmental stress associated with the interplay of poverty, conflict and political violence in the South-south region [43]. The proportion of households affected by conflict and violence in Nigeria’s South-south region steadily increased from 2010 to 2016 [44]. In conflict-prone settings, poor social situations among vulnerable populations such as internal displacement, unemployment, and uncertain livelihood might increase the likelihood of tobacco use although the association between tobacco use and conflict is inconclusive [17, 45]. Other reasons for the regional variation are not clear and would need further qualitative research. Nonetheless, our findings should inform targeted health literacy to increase awareness of the harmful effects of cigarette smoking on human health and improve women’s access to information on tobacco health risks and smoking cessation support services in South-south Nigeria [4, 9, 46].

This study’s finding that being formerly married (divorced/separated/widowed) increased the likelihood of smoking and smoking every day among women is supported by evidence of higher odds of smoking among formerly married women from Ethiopia, Kenya and Iran [18, 27, 47]. In contrast, a prior study found that being married constituted a risk factor for smoking among women in Iran [5], where intrafamily conflicts, show-off and independence, and gender-equality symbols are associated with cigarette smoking among women [46]. The finding of the current study is unsurprising because women who do not have spousal support are more likely to experience social isolation and substantial psychological stress, which often results in cigarette smoking as a coping strategy [48]. Moreover, divorced, separated, or widowed women are far more likely to be in poverty than men with the same marital status, resulting in a high burden of psychosocial stress [43]. Given the rising trend of divorce and separation and prevalent negative widowhood practices in Nigeria [49], cigarette smoking prevention and cessation interventions must be tailored to the preferences and concerns of divorced, separated, and widowed women.

Relatedly, we found that women from female-headed households were more likely to smoke than those from male-headed households. A prior Swedish study also found that women living in single households were more prone to smoke [29]. This study, to our knowledge, is the first to examine the influence of female-headed households on smoking in sub-Saharan Africa. Two reasons might account for our finding. First, it appears that the women from female-headed households in our study may have smoked cigarettes to cope with negative feelings associated with social isolation or disconnection, which has been identified as a major risk factor for detrimental health behaviours [48, 50]. Secondly, female-headed households potentially face a higher risk of poverty and lack of opportunities because of the cultural and social stigmas attached to their marital status [50]. In Nigeria, female heads of households faced a greater chance of poverty than male heads of households after accounting for household size, location, education, employment, and marital status [51]. Since smoking is a coping strategy adopted by women to cope with psychosocial stress resulting from multi-dimensional poverty, ensuring women in female-headed households benefit from Nigeria’s poverty-reduction and social protection initiatives might help address the root cause of cigarette smoking among WRA. Also, cigarette smoking cessation services should be designed to target women in female-headed households.

Ownership of mobile phones was found to increase the risk of smoking and occasional smoking among women in this study. Our finding is contrary to the finding that smartphone owners did not differ from nonowners on the frequency of smoking in a preceding study [52]. The increased odds of smoking among women who own mobile phones might be due to the high prevalence of health misinformation on issues related to smoking products on social media platforms [53], given that women often use the internet and mobile applications for health-related purposes [54]. Although, the DHS data did not disaggregate mobile phones into the basic mobile phone (which cannot connect to the internet) and smartphones (which are internet-enabled), our finding highlights an opportunity for deploying smartphone applications to reach women with tailored tobacco prevention and control messages. Nevertheless, low smartphone penetration might limit the application of smartphones for tobacco control. Whereas 78% of women own a mobile phone in Nigeria, only 48% of them own a smartphone [55]. Urban-rural inequities in smartphone ownership, varying from 29.5-42% in rural areas to 58.2-61% in urban areas, further constrain the utilization of smartphones in Nigeria [55, 56]. Therefore, a first step in deploying smartphone applications to reach women with tobacco prevention and control messages would be to improve their ownership of smartphones through a deliberate policy of digital expansion that reduces to cost of smartphones [55, 56]. Furthermore, it is imperative to reduce the cost of broadband services so that women, irrespective of wherever they live or work, can sustain the use of their smartphones [55, 56].

This study contributes to scholarship on determinants of smoking and smoking frequency by analysing data from a nationally representative survey with a large sample, high precision, and generalisability. Nonetheless, our findings might be limited by three factors. First, as this study was a cross-sectional study design, a cause-and-effect relationship cannot be established. Secondly, women may have under-reported their smoking behaviour as self-report has been found to underestimate tobacco use due to social desirability and recall bias. Thirdly, this study assessed only the prevalence of current cigarette smoking at the time of the survey and did not have the lifetime prevalence of cigarette smoking.

## Conclusion

The aim of the study, which is to assess the prevalence and determinants of smoking and smoking frequency among women of reproductive age in Nigeria, is achieved. The prevalence rates of cigarette smoking, daily smoking and occasional smoking among Nigerian women are low. Overall, women aged 25-34, residing in the South-south region, being formerly married, in female-headed households and owning mobile phones were more likely to smoke. Being formerly married, and in a female-headed household predisposed to daily smoking, while age 15-24 was protective of daily smoking among women. Ownership of mobile phones increased the odds of occasional smoking among women. Women-centred approaches to tobacco prevention and cessation must incorporate these determinants into interventions targeting women of reproductive age in Nigeria.

## Data Availability

The data used for this study are from the 2018 Nigeria Demographic and Health surveys (NDHS) and are publicly available here: https://dhsprogram.com/data/available-datasets.cfm Data was accessed by the researchers upon registration.

## Abbreviations

DALYs: Disability-adjusted life-years
EA: Enumeration area
FMOH: Federal Ministry of Health
HIC: High-income countries
LGA: Local government area
LMICs: Low- and middle-income countries
MAN: Manufacturers Association of Nigeria
NATOCC: National Tobacco Control Committee
NDHS: Nigeria Demographic and Health Survey
NHREC: National Health Research Ethics Committee
NPHC: Nigeria’s Population and Housing Census
SON: Standard Organization of Nigeria
VIF: Variance inflation factors
WHO: World Health Organization
WRA: Women of reproductive age
